# Protocol of a multicenter, single-blind, randomized, parallel controlled trial evaluating the effect of microbiological rapid on-site evaluation (M-ROSE) guiding anti-infection treatment in patients with severe hospital-acquired pneumonia

**DOI:** 10.1186/s13063-023-07570-z

**Published:** 2023-08-23

**Authors:** Xiuli Wang, Kaifei Wang, Fei Xie, Zhihai Han, Yuhong Liu, Lei Pan, Guangfa Zhu, Zhixin Cao, Peng Yan, Li Xiao, Zhimei Duan, Ye Hu, Kun Xiao, Xuxin Chen, Han Fu, Yinghan Shi, Yuwei Song, Xiaobo Han, Wuxiang Xie, Lixin Xie

**Affiliations:** 1 College of Pulmonary and Critical Care Medicine, Chinese PLA General Hospital, Beijing, China; 2 Chinese PLA Medical School, Beijing, China; 3 Department of Respiratory and Critical Care, Beijing Shijitan Hospital, Capital Medical University, Beijing, China; 4 Department of Respiratory and Critical Care Medicine, Beijing Anzhen Hospital, Capital Medical University, Beijing, China; 5 Department of Respiratory and Critical Care Medicine, Beijing Institute of Respiratory Medicine and Beijing Chao-Yang Hospital, Capital Medical University, Beijing, China; 6 Department of Respiratory and Critical Care Medicine, AMHT Group Aerospace 731 Hospital, Beijing, China; 7 Peking University Clinical Research Institute, Peking University Health Science Center, No. 38 Xueyuan Road, Haidian District, Beijing, 100191 China

**Keywords:** M-ROSE, Severe hospital-acquired pneumonia, mNGS, Drug-resistance bacteria, Individual antibiotic treatment

## Abstract

**Introduction:**

The mortality rate of hospitalized patients with severe hospital-acquired pneumonia (SHAP) remains high. Empirical broad-spectrum antibiotic coverage and the misuse of high-grade antibiotics could lead to the emergence of multi-drug and even pandrug-resistant bacteria. In addition to metagenomic next-generation sequencing (mNGS), microbiological rapid on-site evaluation (M-ROSE) might be a useful technique to identify the pathogens in the early stage; however, the effect of M-ROSE guiding anti-infection treatment on prognostic outcomes of SHAP patients is still unclear.

**Methods/design:**

This is a multicenter, single-blind, prospective, randomized controlled trial to evaluate the effect of M-ROSE guiding anti-infection treatment in SHAP patients, which will provide new strategies for the prevention and control of clinical multi-drug resistance bacteria. A total of 166 patients with SHAP, aged 18 years and over, will be recruited from seven centers in Beijing and randomly assigned to the intervention group (M-ROSE combined with mNGS) or the control group (mNGS only) in a 1:1 ratio using the central randomization system. Patients in the intervention group will accept M-ROSE and mNGS analysis, and the control group will accept mNGS analysis. Individualized anti-infective treatment and routine treatment will be selected according to the analysis results. The primary outcome is the ICU outcome (mortality). The safety of the intervention measures will be evaluated during the entire trial period. This trial will be the first randomized controlled trial to evaluate the effect of M-ROSE guiding treatment on mortality in patients with SHAP and may change the prevalence of multi-drug resistant bacteria.

**Ethics and dissemination:**

This trial adheres to the Declaration of Helsinki and guidelines of Good Clinical Practice. Signed informed consent will be obtained from all participants. The trial has been approved by the Chinese PLA General Hospital (Approval Number: 20220322001).

**Trial registration:**

ClinicalTrials.gov NCT05300776. Registered on 25 March 2022.

**Supplementary Information:**

The online version contains supplementary material available at 10.1186/s13063-023-07570-z.

## Strengths and limitations of this study

- This trial will be the first randomized controlled trial to evaluate the effect of microbiological rapid on-site evaluation (M-ROSE) on mortality among patients with severe hospital-acquired pneumonia (SHAP).
- This trial may demonstrate an applicability and efficiency detecting technique to recognize early infection and dynamic monitoring of disease changes of SHAP.
- The anti-infection regimen referring to the authoritative guidelines and combining with the actual situation of local people.
- We may investigate the microbiome data of multi-drug bacteria with high frequency and might find additional link between drug resistance genotypes and phenotypes.

## Introduction

Hospital-acquired pneumonia (HAP) is the most common nosocomial infection and severe hospital-acquired pneumonia (SHAP) is a major risk factor for mortality of hospitalized patients with a mortality rate as high as 50% [1]. Compared with community-acquired pneumonia, HAP has significant differences in pathogenicity spectrum, the risk of potential multidrug-resistant bacterial infection, and the selection of antibacterial drugs. In China, common pathogens of SHAP include *Acinetobacter baumannii*, *Pseudomonas aeruginosa*, *Klebsiella pneumoniae*, *Staphylococcus aureus*, and *Escherichia coli *[2]. Therefore, rapid on-site etiological diagnosis is the key to improve the current extensive use of empirical antibiotics and guide individualized treatment. Traditional clinical pathogen detection methods suffer from limitations in sensitivity, specificity, expediency, and amount of information and are poor at rapidly identifying unknown or rare pathogenic microbes. With the development of metagenomic next-generation sequencing (mNGS) technologies lately, pathogen detection techniques have undergone significant improvement which could enable direct, high-throughput sequencing of nucleic acids from clinical samples. By performing comparative analyses with biological information, the types and abundance of pathogenic microbes contained in the samples can be determined based on a comparison of the database results, which facilitates the identification of large numbers of pathogenic microbes (including viruses, bacteria, fungi, and parasites) [3–6]. A recent study demonstrated that the diagnosis of lower respiratory tract infections and identification of antibiotic-resistant genes can be achieved with high sensitivity and specificity within 6 h using the latest Oxford nanopore metagenomics technology [7]. NGS now plays an important role in the diagnosis of known and unknown pathogenic microorganisms, such as severe acute respiratory syndrome coronavirus 2 (SARS-CoV-2) and Scedosporium apiospermum [8, 9]. In addition, retrospective studies have demonstrated the obvious advantages of mNGS for the detection of community-acquired pathogens [10]. However, the challenge in differentiation among infection, colonization, and contamination greatly reduces the diagnostic value of mNGS. We still urgently need a new rapid etiological diagnosis technology to break this vicious cycle.

Rapid on-site evaluation (ROSE) includes cytological ROSE (C-ROSE) and microbiological ROSE (M-ROSE) [11]. C-ROSE is often accompanied by the sampling process for on-site cytological interpretation and is widely used in the diagnosis of respiratory tumor lesions. M-ROSE is a microbial etiological examination of lower respiratory tract specimens that also requires cytological evaluation, which can be applied at the bedside in the ICUs for rapid pathogenic diagnosis of lower respiratory tract infections. The main step of M-ROSE is to perform Diff-Quik staining and Gram staining of the lower respiratory tract specimens for microscopic observation and interpretation. It determines the origin of specimens through the presence of many kinds of cells. It also determines the presence of an infection in a specimen and possible pathogens by examining the proportion of neutrophils, number of pathogens of the same genus in the field of view, and phagocytosis of neutrophils, thereby suggesting bacterial and fungal infections. The contaminated sample always distributes many bacteria and more squamous epithelial cells, fewer or no inflammatory cells. Conversely, the sample of infection will have high-quality, less or no cell, and colonized bacteria of the upper respiratory tract, and a certain amount of inflammatory cells, and may exist neutrophil phagocytosis. Therefore, we assume that combining M-ROSE with the mNGS for rapid and accurate diagnosis of the SHAP causative agent is of great significance.

On the other hand, the emergence of antibiotic-resistant bacteria is also an important obstacle in the clinical treatment of SHAP mainly due to the inappropriate use of antibiotics [12]. Studies have shown that the precise dose of antibiotics can not only provide a sufficient drug concentration to achieve clinical treatment effects but also minimize bacterial toxicity and drug resistance development [13]. Tracking the antimicrobial-resistant bacteria, analyzing the homology, and traceability are of great clinical significance. Relevant studies have found that the molecular characteristics of carbapenem-resistant *Enterobacteriaceae* in hospitals and their phylogenetic relationships can be successfully determined through sequencing technologies based on multiple platforms, and the source of their outbreaks can be traced based on this strategy [14]. Through combination of epidemiological data and phylogenetic tree analysis, it was determined that *Flavobacterium* may be the ancestral source of the tigecycline resistance gene tet (X) and its corresponding transmission mechanism [15]. In addition, the clinical evolutionary route of carbapenem-resistant and highly virulent *Klebsiella pneumoniae* was discovered through whole-genome sequencing and bioinformatics analysis [16]. Furthermore, many countries have integrated genome sequencing into antimicrobial resistance monitoring systems [17–19]. Consequently, we attempt to establish a standard clinical analysis framework to track the source of antimicrobial-resistant bacteria in hospitals and determine their transmission routes.

## Method

### Study design

**Fig. 1:**
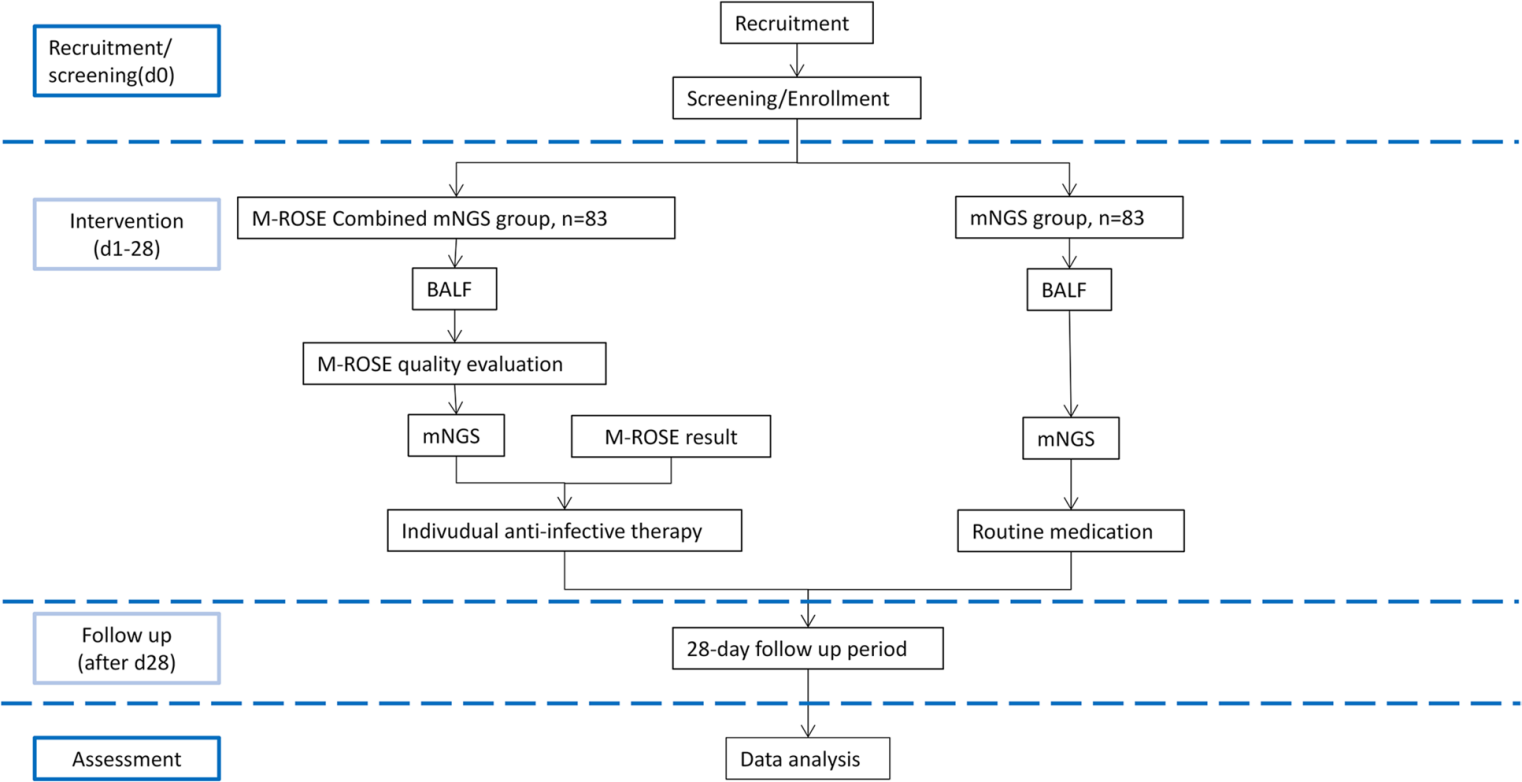
The flowchart of the trial

This study is a multi-center, single-blind, randomized, controlled superiority trial conducted at seven medical centers in tertiary general hospitals among patients with SHAP. The primary hypothesis is that compared with mNGS alone, M-ROSE combined with mNGS by guiding the rational use of antibiotics can get greater attainment to reduce the mortality of SHAP patients. The secondary hypothesis is that M-ROSE combined with mNGS can lessen hospital admission rates, length of stay, and drug resistance of SHAP patients. A total of 166 participants will be enrolled and randomly assigned in a 1:1 ratio to either the intervention group (M-ROSE combined with mNGS analysis group) or the control group (mNGS analysis group) using the central randomization system. After tracheoscopy, the intervention group will accept the antibiotic treatment scheme under the guide of M-ROSE and mNGS, following an anti-infective treatment regimen based on Management of Adults With Hospital-acquired and Ventilator-associated Pneumonia by the IDSA/ATS and guided by the local teams of internists, emergency physicians, pulmonologists, infectious disease specialists. The control group will be provided routine anti-infective treatment based on clinical experience according to mNGS. The course of both groups’ medications was continued for at least 7 days, then following up the ICU outcomes and other secondary outcomes. The research flowchart is shown in Fig. 1. The protocol for this study has been developed based on the Standard Protocol Items: Recommendations for Interventional Trials (SPIRIT) checklist (Additional file 1).

### Inclusion criteria and exclusion criteria

In this trial, according to the guidelines of domestic and foreign [2, 20], pneumonia is defined by a chest X-ray or computed tomography (CT) image showing a new or progressive infiltrative shadow, consolidation shadow, or ground-glass shadow plus two or more of the following three clinical syndromes: (1) fever and body temperature > 38 °C, (2) purulent airway secretions, and (3) peripheral blood leukocytes > 10 × 10^9^/L or < 4 × 10^9^/L.

HAP is defined as new pneumonia that occurs 48 h after admission in a patient that has not undergone invasive mechanical ventilation during hospitalization and is not in the incubation period of pathogenic infection.

Ventilator-associated pneumonia (VAP) is a special type of HAP that is defined as pneumonia that occurs after 48 h of mechanical ventilation in patients with tracheal intubation or tracheotomy or within 48 h after weaning from mechanical ventilation or extubation; VAP is also included in HAP.

According to the diagnostic criteria of SHAP, patients who meet one of the following criteria will be included: (1) the need for tracheal intubation and mechanical ventilation and (2) vasoactive drugs after aggressive fluid resuscitation for infectious shock.

Patients meeting any of the following criteria will be excluded from the trial:

1. Patients without bronchoalveolar lavage (BAL)
2. Patients without bronchoalveolar lavage fluid (BALF) specimens for mNGS etiological examination
3. Patients aged < 18 years old
4. Patients expected to be hospitalized for less than 3 days
5. Patient with long-term mechanical ventilation (> 60 days)
6. Those who do not agree to be included in this study

### Participant recruitment

Participants will be recruited from seven medical centers in tertiary general hospitals in North China including Chinese PLA General Hospital First Medical Center, Chinese PLA General Hospital Sixth Medical Center, Chinese PLA General Hospital Eighth Medical Center, Beijing ShiJiTan Hospital, Beijing AnZhen Hospital, Beijing Chao-Yang Hospital, and AMHT Group Aerospace 731 Hospital.

Each branch center will post and distribute printed recruitment posters inside and outside the hospital. Patients with SHAP will be invited to participate in this study after visiting the branch center. Patients who meet the inclusion criteria and provide signed informed consent will enter the screening period. At the screening visit, a trained research staff (physicians) will confirm the eligibility by the symptom, laboratory result, and chest X-ray or CT image and check every inclusion and exclusion criterion. Patients who meet the exclusion criteria will be excluded from this study within 24 h. The recruitment time is 24 months, from July 2022 to June 2024.

### Randomization

Qualified participants will be randomly assigned 1:1 to the intervention group and control group by a hierarchical center randomization procedure based on the network stochastic system, stratified according to the center. When the sub-center includes qualified participants, researchers can log in to the network random system for random allocation. The central randomization system will then assign an identification code (SSID, 4 bits) and a random number (9 bits) for each participant. Once a random number has been assigned to a subject, it cannot be assigned again. Subjects who failed to complete the entire study could not be replaced.

### Blinding

The patients and research personnel who are responsible for laboratory examination, data collection, end-point evaluation, and statistical analysis will be blinded to the allocation of the two intervention arms. However, it is impractical to blind the attending physicians due to the nature of antibiotic intervention. Therefore, clinicians and researchers will not be blinded to the treatment allocation.

### Intervention

As mentioned in the introduction, M-ROSE may benefit the diagnosis and treatment of patients with SHAP. So far high-quality studies of the effects of M-ROSE in multicenter randomized controlled trials are lacking. Therefore, this trial is needed to evaluate the value of M-ROSE combined with mNGS in patient-individualized anti-infective therapy. After fully evaluating indications and contraindications, both the intervention group and control group will undergo BAL and mNGS detection at least once.

For the intervention group, the quality of BALF will be evaluated by M-ROSE and the qualified or good specimens will be screened and submitted for mNGS examination. Next, we will analyze the infecting pathogen by morphological feature during 1–2 h and provide an M-ROSE analysis report to physicians. Then, antibiotic treatment will be selected according to the recommendations of an individualized anti-infection regimen based on the etiological results of the first M-ROSE analysis. At 48–72 h after enrollment, the mNGS results will be published, and we will perform the second M-ROSE analysis. According to the comprehensive results of M-ROSE, mNGS, and clinical pathogenic examinations (bacterial smear, culture, etc.), the anti-infective treatment will be determined to continue or adjust. The third M-ROSE analysis will be performed on days 3–5 after enrollment. The above process will be repeated. The course of antibiotic treatment should be ≥ 7 days. In principle, no more than 5 M-ROSE analyses will be performed for each patient until the primary outcome.

The anti-infective treatment regimen is based on Management of Adults With Hospital-acquired and Ventilator-associated Pneumonia by the IDSA/ATS and guided by the expert committee of internists, emergency physicians, pulmonologists, and infectious disease specialists. Throughout the study, concomitant medications or treatments deemed necessary will be prescribed. See Table 1 for specific regimen of pathogens and the approach and dose to achieve this treatment.

**Table 1:** Individualized anti-infection regimen

Pathogen	Antibiotic regimen	Drug	Dose	Frequency	Approach
*Staphylococcus aureus*	Linezolid + meropenem	Linezolid	0.6 g	q12	Intravenous drip
		Meropenem	1 g	q8	Intravenous pumping
	Vancomycin + meropenem	Vancomycin	First 1 g then 0.5 g	q8	Intravenous drip
		Meropenem	1 g	q8	Intravenous pumping
*Klebsiella pneumoniae*	Tigecycline + meropenem	Tigecycline	First 0.1 g then 0.05 g	q12	Intravenous drip
		Meropenem	1 g	q8	Intravenous pumping
	Colistin + meropenem	Colistin	50w	q12	Intravenous drip
			25w	q12	Aerosol inhalation
		Meropenem	1 g	q8	Intravenous pumping
*Acinetobacter baumannii*	Sulbactam and cefoperazone + meropenem	Sulbactam and cefoperazone	3 g	q8	Intravenous pumping
		Meropenem	1 g	q8	Intravenous pumping
	Colistin + Sulbactam and cefoperazone + Meropenem	Colistin	50w	q12	Intravenous drip
		Sulbactam and cefoperazone	25w	q12	Aerosol inhalation
			3 g	q8	Intravenous pumping
		Meropenem	1 g	q8	Intravenous pumping
*Pseudomonas aeruginosa*	Piperacillin tazobactam + levofloxacin	Piperacillin tazobactam	4.5 g	q8	Intravenous pumping
		Levofloxacin	0.5 g	qd	Intravenous drip
	Colistin + meropenem	Colistin	50w	q12	Intravenous drip
			25w	q12	Aerosol inhalation
		Meropenem	1 g	q8	Intravenous pumping

The notice of linezolid includes blood platelet > 100 × 10^9^/L; the notice of vancomycin includes blood concentration: 10 ~ 20mg/L

For the control group, the BALF will be directly sent for mNGS examination, and routine anti-infective treatment will be provided firstly based on clinical experience. At 48–72 h after enrollment, the mNGS results will be published, according to the comprehensive results of mNGS and clinical pathogenic examinations (bacterial smear, culture, etc.), the anti-infective treatment regimen will be determined to continue or adjust.

During the follow-up period, if a patient’s condition is found to be aggravated by M-ROSE analysis, clinical etiological examination results, or clinical symptoms, a meeting of the expert committee will need to be convened to discuss the patient’s antibiotic treatment plan after an investigation. In order to improve adherence to interventions, when patients refuse to use expensive antibiotics, they can be treated under the guidance of drug sensitivity results. Besides, we organize personal assessments and guidance of research investigators and associated personnel every 2 weeks. In the event of missed visits, study investigators will contact participants by phone and inquiry about barriers to participation.

### Measurements and data collection

The formal baseline data collection includes demographic data (hospitalization number, name, sex, age, main cause of admission, ethnicity, smoking history, body mass index); history of diseases (hypertension, coronary heart disease, diabetes, kidney disease, hepatopathy, cerebrovascular disease, autoimmunity disease, hemopathy, pulmonary disease, and cancer); medication use ( hormonotherapy and vasoactive agent); physical examinations (blood pressure, pulse rate, heart rate, respiratory rate, temperature); the way of oxygen therapy (nasal catheter oxygen inhalation, oxygen mask, high-flow oxygen therapy, noninvasive mechanical ventilation, invasive mechanical ventilation); and the severity scores including the Acute Physiology and Chronic Health Evaluation II (APACHE II) and the Sequential Organ Failure Assessment score (SOFA).

The case report form (CRF) has been designed by the study team referring to standard questions from previous studies [21, 22]. The information collector will visit sub-center to input required data including baseline data, laboratory results, and outcomes in Data Management System (DMS) every week after new participant enrollment.

BALF or endotracheal aspirate (ETA) samples collections and detecting: participants will accept bronchoscopy by trained respiratory physician to acquire lower respiratory samples in 24 h. Samples will be frozen then transported through complete cold chain and measured in Beijing Hugobiotech Health Laboratory. The optimized MicroExtract nucleic acid extraction process is employed to extract microbial nucleic acids from bacteria, fungi, viruses, and parasites present in the samples. Additionally, the PACEseq analysis process, which is based on artificial intelligence automation, is utilized to analyze the genomic data. For the intervention group, BALF will be processed by centrifuging, dropping the supernatant, and mixing. Then, an appropriate amount of specimen will be spread on the slides equably, drying, fixing, and staining by gram and diff stain. After the above, trained researchers will observe the slides under the microscope, evaluate the quality by classifying and counting cells, and recognize pathogenic including bacteria, fungi, and parasites. This course will be executed by microorganism-related-trained physicians of each sub-center. Every BALF testing result (M-ROSE and mNGS) will be sent to physicians in order to guide the treatments.

Antibiotic therapy: for the intervention group, attending physicians will be required to follow an antibiotic regimen and be monitored by the Clinical Research Associate (CRA) and other researchers. In order to conform to reality, we offer 2 regimens to doctors for selection. The regimens have been adapted by local teams of internists, emergency physicians, pulmonologists, infectious disease specialists, and clinical epidemiologists (the details are listed in Table 1). If the antibiotic regimen is reversed, the research center must be notified as soon as possible. During the study, if the microbial susceptibility results is clear, physicians can replace antibiotic and inform to researchers. Besides, the dose adjustment of individual patients is appropriately judged by the attending physicians and reported to the expert committee for discussion. We will also record and report antibiotic use condition every week to improve the trial compliance.

Traceability of drug-resistant bacteria: The mNGS data of the samples were analyzed to compare drug-resistance gene detection with the results of were corresponding to the results of clinical culture and drug sensitivity. Selecting the high-frequency multi-drug-resistant bacteria to sequence by Whole Genetic sequence (WGS) to obtain the multilocus sequence typing (MLST) typing and phylogenetic tree.

### Follow-up schedules

The physicians will report the primary outcome when patients reach the ICU outcome (discharge or death). Then the 28-day mortality, the length of stay in ICU/hospitalization, and other outcomes will be recorded by the data collectors. Besides, the inflammatory indicators within 2 weeks, M-ROSE results, mNGS results, clinical etiology test results, the main clinical symptom, the feature of medical imageology, the volume of hydrothorax, and antibiotics used condition will be recorded weekly during the trial. The medication use will also be monitored, if a participant’s condition occurs worse or serious adverse events, an expert meeting will be convened to adjust the therapy and be documented. Various parameters are followed up according to the data collection time points and the schedule of measurements and collection of this trial is summarized in Table 2.

**Table 2:** The schedule of measurements and visits of this trial

	Screening	Intervention^a^							Follow-up	
Unit: day	1	2	3	4	5	6	7	8	14	28
Informed consent form	√									
Inclusion/exclusion criteria	√									
Get random number	√									
Demographic data		√								
History of diseases		√								
Medication use		√								
Physical examinations		√								
Way of oxygen therapy		√								
APACHE II scores		√								
SOFA scores		√								
Clinical symptom		√								
M-ROSE analysis		√		√		√				
mNGS analysis		√								
Inflammatory indicators		√		√		√		√	√	
Clinical pathogen detection		√					√		√	
Antibiotic use record^a^										
Length of stay in ICU/hospitalization										√
Primary outcome										√
28-day survival follow-up										√

*APACHE* Acute physiology and chronic health evaluation, *SOFA* Sequential Organ Failure Assessment

^a^Antibiotic use record will be collected on the daily basis

### Data management

Researchers of various roles involved in clinical trials, such as investigators, study coordinators, site monitors, and data managers, can enjoy unique benefits offered by electronic data capture (EDC). We use the Web-based Data Management System (DMS) based on the CRF to facilitate data collection and central management during the whole process of the trial. The acquisition librarian is responsible for the collection of data monitored by independent external supervisors. Questions or outliers in the original questionnaire shall be answered by the quality control officer. Randomly select 10% of the subjects, test them with the original questionnaire, evaluate their bit error rate, and arrange the next monitoring plan according to the bit error rate. If there is an error, the erroneous data in the database must be corrected and a record of the modification of the database must be kept in the electronic data collection system. Lock database and data review. After data input and troubleshooting, the research data will be frozen, and the researcher cannot change the data to ensure the stability of the data. If the principal investigator verifies that the frozen database has no data problems, the database will be locked. The post-trial care of the patients will be followed up until hospital discharge. No compensation will be provided.

### Outcomes

The primary outcome is ICU outcomes (mortality). The secondary outcomes include 28-day mortality; ventilator-free days and ICU-free days; body temperature changes; inflammatory indicators including leukocyte count, neutrophil ratio, interleukin 6 (IL-6), C-reactive protein (CRP), and procalcitonin (PCT) levels within 2 weeks of admission; M-ROSE results (quality and infectious pathogens); mNGS results (pathogen gene sequencing and bacterial drug resistance gene test results); clinical etiology test results (specimen smear, bacterial and fungal culture of the specimens); the main clinical symptom (cough, abundant phlegm, dyspnea, hemoptysis, fever); the feature of chest X-ray or computed tomography (CT) image; the volume of hydrothorax; length of hospitalization; antibiotics used condition (type, dose and duration); and source of drug-resistant bacteria.

### Sample size

In this study, a randomized controlled trial design is used to evaluate the treatment effect of M-ROSE in patients with severe hospital-acquired pneumonia. The primary outcome is ICU hospitalization clinical outcome (mortality). We assumed that *α* = 0.05 (two-sided test), *β* = 0.20, and 28-day mortalities among the mNGS group and M-ROSE plus mNGS group will be 70% and 48%, respectively, according to the results of relevant research [23] and our previous study [24]. Seventy-five patients should be recruited in each group. Considering an attrition rate of 10%, the eligible participants in each group should be 83. Therefore, we determined that the sample size should be 83 in each group (*n* = 166 in total).

### Statistical analysis

The primary analyses will follow the intention-to-treat principle and will be conducted among participants who have been randomized. Per protocol analysis will be performed as sensitivity analysis among those hospitalized for ≥ 3 days after randomization. For the outcome of dichotomous variables, we will use the Cox regression models to obtain the hazard ratio and its 95% confidence interval, which will be used to evaluate the efficacy of M-ROSE guiding treatment. For repeatedly measured continuous outcome, we will use linear mixed models to compare the differences between the intervention group and the control group. For the survival analysis of the primary outcome, we will plot the survival curve, calculate the survival rate using the Kaplan–Meier method, and compare the existence of statistical significance between the survival curves using the log-rank test. Continuous data will be expressed as the mean ± standard deviation or median and interquartile range (skewed distribution). The difference between groups will be compared using a *t* test or nonparametric test (skewed distribution). Categorical data will be expressed as a percentage (%), and groups will be compared using the chi-square or Fisher exact tests. For the primary outcome and important secondary outcomes, we will conduct subgroup analyses to explore whether the presence of effect modifiers will affect the efficacy of M-ROSE-guided treatment. Potential modifiers of the intervention effect include age, sex, invasive mechanical ventilation, disease severity indicators, and underlying diseases. SAS 9.4 statistical software will be used for all analyses in this project, and the significance level of the statistical test will be set to 0.05 (double-sided). Per-protocol analyses will be conducted among participants including those who will accept treatment more than 3 days with the guide of study detection methods and completed the final follow-up. Subgroup analyses will be performed to identify potential modifiers of the intervention effect, including gender, age, ethnicity, smoking history, body mass index, history of diseases, inflammatory indicators, antibiotics use, the main clinical symptom, clinical etiology test, the feature of chest X-ray or computed tomography (CT) image, the way of oxygen therapy, and the severity scores. All available SPSS applications will be used to check/analyze data integrity. When analyzing the data, if missing data are found, we will first contact the data collectors to supplement missing data and analyze the reasons if possible. Missing values were filled in by the last data. For example, baseline data will be filled in for participants who do not have 28-day follow-up visit. The reasons for withdrawing from the study are recorded in the data file. Participants with no or incomplete primary outcome data (28-day mortality, ICU mortality) will be excluded from the analysis. When participants reach 30% of recruitment, a first interim analysis will be conducted to determine whether there are similarities between the two groups of demographic statistics (number of hospitalizations, name, gender, age and main reason for hospitalization, race, history of smoking, body mass index). A second interim analysis will be undertaken to analyze whether difference in the results. When follow-up data of participants reach 50%, The results will guide decisions regarding the continuation of the study.

### Ethics and dissemination

This trial adheres to the Declaration of Helsinki and guidelines of Good Clinical Practice. The subjects or legal representative will receive sufficient explanation and sign the informed consent (Additional file 2) form prior to the study. Participant data in the DMS will be protected by password and only available to users designated by the study with appropriate authorization levels. De-identified data will be used for statistical analysis. The trial has been approved by the Chinese PLA General Hospital (Approval Number: 20220322001).

The results of this trial will be disseminated through academic conferences and publications in international peer-reviewed journals.

### Quality control

Before the study begin, the principal investigator accepted good clinical practice (GCP) training and all researchers accepted training including study protocol, informed consent, case report form, standard operating procedures of participants’ data collections, collection and preservation methods of biological samples to ensure the trail quality. Besides, we have established a detailed investigator manual to ensure compliance with the protocol.

During the study, biological samples will be tested by trained specialists and genetic testing company. In addition, 10% of BALF samples will be taken as split samples to control the quality of M-ROSE test results.

The design and execution of multi-center clinical trials require the joint efforts of all established departments including expert committee composed of clinicians, statisticians, and quality management personnel to determine the methods of clinical trials and solve major problems in practical applications.

Executive committee: a group composed of expert groups members. The executive committee is responsible for clinical trials, organizing seminars related to trials, and data management and analysis. If necessary, executive committee conferencing will be convened for quality control.

Quality control team: consists of CRA, who will investigate and monitor the research every 2 weeks by checking the integrity and informed consent, criteria for inclusion and exclusion and the original data, treatment of AEs and SAEs, sample storage conditions, and records of data collection and offer reports to PI and committee. Adverse events, such as injurious falls, cardiac events, medication side effects, or death, will be recorded using data collected for secondary outcomes such as mortality and hospitalization. Once an adverse event is announced, participating facilities will be required to report it immediately to the quality control department. If the event is considered serious, the cause and prognosis are confirmed by the principal investigator or other clinical members of the trial management team. All incidents will be followed until the incident is resolved or decided not to be pursued. All relevant information will be shared between the researchers. Besides, any modification to the protocol, including study types, study design, subjects, sample sizes, or test procedures, should make a formal protocol amendment and get approval of the Ethics Committee; details will be updated in ClinicalTrials.gov. Researchers may need to regain recipients’ informed consent if design changes may affect the testing risks.

## Trial status

Recruitment began July 6, 2022, and we anticipate recruitment will be completed in December 2024. The version identifier: V 1.0; Version date: 28 Sep 2022.

## Patient and public involvement

No patients or the public were involved in the design, conduct, reporting, or dissemination of this research study.

## Discussion

HAP is one of the most common nosocomial infections [25], which can lead to a significant increase in the length of hospitalization, hospitalization costs, and mortality of critically ill patients [26, 27]. Studies have shown that the ICU has a high incidence of nosocomial infections. Approximately 19.2% of ICU patients have infections, while 5.2% of patients in other hospital wards have infections [28, 29]. After 48 h of mechanical ventilation, VAP is expected to affect 10–25% of all ventilated patients [30]. Therefore, the prevention and management of nosocomial infections, especially HAP, in critically ill patients present challenges, the first of which is the early diagnosis of microbial etiology. In addition, antibiotic-resistant bacterial infections are an urgent problem in the clinical setting. Improper use of antibiotics has led to the emergence of antibiotic resistance at an alarming rate, and it has been recognized as the major healthcare challenge of the century because the occurrence and dissemination of drug-resistant bacteria can dramatically increase the risk of death in critically ill patients [31, 32].

In recent years, the emergence of metagenomic sequencing technology has helped in the diagnosis of SHAP [33]. However, there remain shortcomings in terms of efficiency and pertinence. The emergence of M-ROSE undoubtedly provides a new possibility for the rapid diagnosis of etiology at the bedside. As a novel pathogenic diagnostic technique, the combination of mNGS not only allows for efficient and cost-effective pathogenic diagnosis but also may improve individualized antibiotic guidance for patients with SHAP and reduce the occurrence of drug resistance in clinical practice.

The advantages of this study are as follows. For the first time, a large-scale, multicenter, blinded, randomized design and strict quality control trial will be used to evaluate the effect of M-ROSE-guided individualized anti-infective therapy on 28-day mortality in patients with SHAP. Furthermore, this trial will also investigate the predictive value of rapid drug resistance gene detection on the sensitivity of pathogenic microorganisms. Drug-resistant bacteria will be traced, and application will be dynamically observed. This study may provide novel ideas for the rational application of individualized antibiotics and the prevention and control of drug-resistant bacteria. Nevertheless, this trial will have potential limitations. Because physicians need to treat patients based on the results of M-ROSE and/or mNGS etiological analysis, they cannot be blinded. Single-blind design might lead to performance bias during this trial.

### Supplementary Information

**Additional file 1.** SPIRIT checklist.

**Additional file 2.** Informed consent.

## Data Availability

Not applicable.
