# Supplementary material for: Protocol of a multicenter, single-blind, randomized, parallel controlled trial evaluating the effect of microbiological rapid on-site evaluation (M-ROSE) guiding anti-infection treatment in patients with severe hospital-acquired pneumonia
Source: Trials. 2023 Aug 23;24:552. doi: 10.1186/s13063-023-07570-z (PMC10464107; doi:10.1186/s13063-023-07570-z)
Supplement: Supplementary file 2 — Additional file 2. Informed consent. [file 13063_2023_7570_MOESM2_ESM.pdf]

Additional file 2：Informed consent

Informed consent

(Version number: V1.0 Version date: May 15, 2022)

Dear volunteer subjects or legal representatives:

We invite you to participate in a multicenter prospective clinical trial of M-ROSE (microbiological rapid on-site evaluation) combined with metagenomic sequencing to guide the individualized anti-infective treatment and the prevention and control of drug-resistant bacteria in severe hospital-acquired pneumonia. The study will be conducted in the Department of Chinese PLA General Hospital First Medical Center and other participating units. This study has been reviewed and approved by the Medical Ethics Committee of the PLA General Hospital.

Before you decide whether you want to participate in the study, it is important to understand why the study is being conducted and what it means to participate. Please read the following information carefully and discuss it with friends, family, or your personal physician if you need to. The study physician or research staff will also explain the study to you. You are invited to ask the study physician or study staff any questions you may have before making a decision and signing this informed consent form, and to request more information if needed.

Even if you wish to participate in the study, you may not be eligible to participate. If you are not eligible to participate in the study, the study physician or

study staff will discuss the reasons with you.

Research Background:

Hospital-acquired pneumonia is the most common nosocomial infection in clinical practice. The mortality rate of severe hospital-acquired pneumonia (SHAP) is as high as more than 50%, which is the primary risk factor endangering the lives of hospitalized patients. Due to the difficulty in identifying infection, colonization or contamination by existing clinical techniques, the etiological diagnosis of SHAP is very difficult, which leads to the widespread use of empirical high-level antibiotics, and eventually leads to the occurrence of multi-drug resistance or even all-drug resistance, which seriously affects the survival and prognosis of hospitalized patients with SHAP. Metagenomic next-generation sequencing (mNGS) is an important method for rapid screening of pathogens in clinical practice. However, the lower respiratory tract specimens have low qualification rate, are easily contaminated, and the microorganisms that cannot be identified and detected are infection, colonization or contamination, and inefficiently, which cannot meet the requirements of SHAP anti-infection treatment. Therefore, there is an urgent need for breakthroughs in microbiological rapid on-site evaluation technology.

This team after many years of hard work has been set up beside the bed etiology rapid on-site microbiology evaluation (M-ROSE) system, able to bed lower respiratory specimens were initially determined within half an hour if qualified, by cytological classification, and inflammatory cells within the pathogen screening

preliminary determination of the presence of infection and infection pathogenic agents, fill in the etiology of the bed and rapid evaluation of the blanks. Therefore, the combined application of M-ROSE and mNGS can complement each other and complement each other. Based on the point-of-care rapid pathogen assessment technology (M-ROSE) developed in our previous study, combined with mNGS, this study intends to determine whether the lower respiratory tract specimens are qualified, whether there is infection, which pathogen infection, so as to guide clinical individualized treatment and rational use of antibiotics through a multi-center prospective clinical study. At the same time, new technologies such as third-generation sequencing can trace the source of drug-resistant bacteria, establish a perfect tracking system of drug-resistant bacteria, realize the rapid and accurate treatment of severe pneumonia, alleviate the current situation of multi-drug resistant bacteria infection in the hospital, and improve the survival rate of patients with severe hospital-acquired pneumonia.

Objectives:

(1) About 40 patients(every sub-center) diagnosed with severe hospital-acquired pneumonia were enrolled. After routine bronchoscopy, bronchoalveolar lavage fluid (BALF) samples of 20 patients were sent for metagenomic sequencing (mNGS) and M-ROSE detection. The bronchoalveolar lavage fluid (BALF) specimens of 20 patients were only tested by metagenomic sequencing (mNGS). According to the test results of the two groups, individualized anti-infection treatment and conventional anti-infection treatment were performed respectively. The effect of M-ROSE

combined with mNGS on the mortality of SHAP patients guided by rational application of antibiotics.

(2) About 40 patients(every sub-center) diagnosed with severe hospital-acquired pneumonia were enrolled. Each patient underwent intermittent bronchoscopy according to their condition during hospitalization. The bronchoalveolar lavage fluid (BALF) specimens of 20 patients were sent for metagenomic sequencing (mNGS) and M-ROSE detection. The bronchoalveolar lavage fluid (BALF) samples of 20 patients were tested by metagenomic sequencing (mNGS). According to the results of the two groups, individualized anti-infective treatment and standard anti-infective treatment were performed respectively. The daily changes of inflammatory indicators (including white blood cell count, neutrophil count and proportion, interleukin-6, C-reactive protein, procalcitonin, changes in body temperature, M-ROSE results of lower respiratory tract specimens (whether qualified, whether indicating bacterial infection and infectious pathogens, mNGS of lower respiratory tract) within 2 week of admission were dynamically monitored Results (pathogen gene sequencing results and bacterial resistance gene test results), clinical pathogen test results (specimen smear, specimen bacterial and fungal culture, nucleic acid, etc.), length of hospital stay, 28-day clinical outcome (survival or death), the most antibiotics used at the same time and the time, source of resistant bacteria. And baseline data (hospitalization number, name, gender, age, main reason for admission, APACHE II score, SOFA score, ethnicity, smoking history, BMI, history of lung disease, history of heart disease, history of hypertension, history of diabetes, and other past medical history.

To evaluate the guiding significance of rapid microbiological evaluation (M-ROSE) in the anti-infective treatment of severe hospital-acquired pneumonia.

Range:

Patients hospitalized in the Department of Respiratory Medicine of Chinese PLA General Hospital and participating units

Randomization:

Once enrolled in the study, you may be assigned to the intervention group (M-ROSE plus mNGS etiology analysis group) or the control group (mNGS aetiology analysis alone group), with equal probability of randomization to the two groups, with stratified central randomization using a Web-based randomization system, stratified according to center. Once a random number has been assigned to a subject, the number cannot be reassigned. Subjects who failed to complete the entire study also could not be replaced.

Research overview:

(1) The cases of patients admitted to the Department of Respiratory Medicine of PLA General Hospital and other participating units were used to dynamically monitor the changes of related indicators of patients, and the mortality rate of the two groups was compared to observe whether the primary outcome indicators and secondary outcome indicators were statistically significant.

(2) To evaluate the diagnostic value of rapid microbiological evaluation

(M-ROSE) in severe hospital-acquired pneumonia. Baseline balance analysis between groups: measurement data were expressed as mean ± standard deviation or median and interquartile range (skewed distribution), and the differences between groups were compared by t test or non-parametric test (skewed distribution). Count data were described by percentage (%), and comparison between groups was analyzed by chi-square or Fisher exact probability test, respectively. Effect evaluation analysis: The intention-to-treat analysis principle was used, and all patients who participated in randomization, regardless of whether they dropped out, were required to be included in the final analysis. For binary outcomes, we used Cox regression models with adjustment for study site to obtain HRS and 95% intervals to evaluate the effect of M-ROSE guided therapy as compared with conventional therapy. For outcomes of continuous variables, we will use analysis of covariance to compare the intervention group with the control group, with adjustment for site. For the survival analysis of the primary outcome, we will draw survival curves and use the Kaplan-Meier method to calculate survival rates, and the log-rank test will compare survival curves for statistical differences. Subgroup analysis: For the primary outcome and important secondary outcomes, we will conduct subgroup analysis to explore whether there are any effect modifiers that affect the efficacy of M-ROSE guided treatment. Subgroup stratification factors include: age, sex, invasive mechanical ventilation, disease severity indicators, underlying diseases, etc.All analyses of this project will use SAS9.4 statistical software, and the significance level of statistical test will be two-sided 0.05.

The research program

In this study, the vital signs and laboratory tests during your hospitalization will be counted, and you will be comprehensively evaluated by bronchoscopy intermittently after admission, and each examination time will not exceed half an hour.

Other treatment options

You can choose

■ Do not participate in this study and continue your usual care.

■ Participate in other studies.

Consult with your doctor about your decision.

Possible implications of the study

You may find these tests inconvenient. In addition, some examinations may make you feel uncomfortable. If you have any questions about the tests and procedures used in the study, you can speak to the study physician.

Risks and adverse effects of the study

This study is mainly carried out by means of bronchoscopy, which is safe and

has no allergic reaction and is harmless to human body. You may feel slightly uncomfortable during the process.We will adjust the intensity according to the patient's tolerance. If you experience any discomfort during the bronchoscopy, please

call your study physician in time for consultation.

You will need to tell your family or close friends that you are participating in a clinical study and that they can take note of the events described above.If they have questions about your participation in the study, you can tell them how to contact your study physician.

The benefit

Participation in this study may lead to improvements in your health.

The information obtained from this study will be helpful to assist your diagnosis and treatment process and have a certain warning effect on related complications.

Relevant research information and results obtained from this study will be informed to you in due course.

Reward or compensation

You will not receive any remuneration for participating in the study. In order to compensate you for the inconvenience that may be caused by your participation in this study, the study will pay the cost of relevant examinations performed during your participation in this study.

Medical expenses compensation for injuries caused by the study

If your health does suffer from study-related damage as a result of participating in the study, please notify the study doctor immediately and the study doctor will be responsible for taking appropriate treatment measures.

Even if you have signed this informed consent form, you still retain all your legal rights.If your rights and interests are violated, you can contact the Medical Ethics Committee of the PLA General Hospital,Tel: 010-66937166

Confidentiality

Your medical records will be kept in the hospital and the investigators, research authorities, ethics committees will be allowed access to your medical records. Your personal identity will not be disclosed in any public report of the results of this study. We will make every effort to protect the privacy of your personal medical data within the scope permitted by law.

Personal and medical information about you will be kept confidential and kept in a safe and secure place. At any time, you may request access to your personal information (such as your name and address) and modify it if necessary.

By signing this informed consent form, you consent to the use of your personal and medical information for the purposes described above.

Voluntary

Participation in the study is completely voluntary, and you may refuse to participate in the study or withdraw from the study at any time without any reason. This decision will not affect your future treatment.

If you decide to withdraw from this study, please inform your study physician in advance.In order to ensure your safety, you may be required to undergo relevant

examinations, which is beneficial to protect your health. Please keep this informed consent form.

Subject Consent Statement

By signing below, you confirm that you have read and understood this informed consent form.

I declare:

I had plenty of time to ask questions related to this study and my questions were satisfactorily answered.

I understand that I am voluntarily participating in the study and that I can withdraw from the study at any time without penalty and without any loss of any benefits or medical services to which I am entitled.

My personal health information can be used and passed along as described above and added to the research database. My personal information, medical records, and pathological specimens may be used in future studies other than this study after approval by the ethics committee for the following purposes, including investigators or other companies and individuals working for or with investigators:

An investigational diagnostic technique with greater insight into safety and efficacy was developed.

Patients were studied for other therapies.

A better understanding of the diseases involved in research;And improving the

efficiency, design, and methodology of future clinical studies.

I agree that my personal physician will be informed that I am participating in this study and that they can provide health information about me to the study physician.

I understand that I will not lose any legitimate rights and interests by signing this informed consent form. I will receive a signed and dated copy of the informed consent form. By signing, I agree to participate in the study.

Signature of subjects:__________________ Date:____________

Name (in block letters) :________________ Subject Contact number:___________

Signature of legal representative (if applicable) :_____________Date:___________

Legal representative' name (in block letters):________________

Statement by the investigators

I confirm that the details of the study, particularly the possible risks and benefits of participating in the study, were explained to the patient.

Investigator's signature:___________________ Date:____________

Investigator's name (in block letters) :________Investigator's Contact number:______
